# *Trypanosoma brucei* CYP51: Essentiality and Targeting Therapy in an Experimental Model

**DOI:** 10.1371/journal.pntd.0005125

**Published:** 2016-11-17

**Authors:** Frédéric-Antoine Dauchy, Mélanie Bonhivers, Nicolas Landrein, Denis Dacheux, Pierrette Courtois, Florian Lauruol, Sylvie Daulouède, Philippe Vincendeau, Derrick R. Robinson

**Affiliations:** 1 University of Bordeaux, laboratoire de parasitologie, France; 2 IRD-CIRAD-University of Bordeaux, France; 3 University Hospital of Bordeaux, Department of infectious and tropical diseases, Hôpital Pellegrin, France; 4 University of Bordeaux, Microbiologie Fondamentale et Pathogénicité, France; 5 CNRS, Microbiologie Fondamentale et Pathogénicité, France; 6 Bordeaux INP, ENSTBB, Microbiologie Fondamentale et Pathogénicité, France; 7 University Hospital of Bordeaux, laboratoire de parasitologie, Hôpital Pellegrin, France; Hunter College, CUNY, UNITED STATES

## Abstract

*Trypanosoma brucei gambiense* is the main causative agent of Human African Trypanosomiasis (HAT), also known as sleeping sickness. Because of limited alternatives and treatment toxicities, new therapeutic options are urgently needed for patients with HAT. Sterol 14alpha-demethylase (CYP51) is a potential drug target but its essentiality has not been determined in *T*. *brucei*. We used a tetracycline-inducible RNAi system to assess the essentiality of CYP51 in *T*. *brucei* bloodstream form (BSF) cells and we evaluated the effect of posaconazole, a well-tolerated triazole drug, within a panel of virulent strains *in vitro* and in a murine model. Expression of CYP51 in several *T*. *brucei* cell lines was demonstrated by western blot and its essentiality was demonstrated by RNA interference (CYP51^*RNAi*^) *in vitro*. Following reduction of Tb*CYP51* expression by RNAi, cell growth was reduced and eventually stopped compared to WT or non-induced cells, showing the requirement of CYP51 in *T*. *brucei*. These phenotypes were rescued by addition of ergosterol. Additionally, CYP51^*RNAi*^ induction caused morphological defects with multiflagellated cells (*p*<0.05), suggesting cytokinesis dysfunction. The survival of CYP51^*RNAi*^ Doxycycline-treated mice (*p* = 0.053) and of CYP51^*RNAi*^ 5-day pre-induced Doxycycline-treated mice (*p* = 0.008) were improved compared to WT showing a CYP51 RNAi effect on trypanosomal virulence in mice. The posaconazole concentrations that inhibited parasite growth by 50% (IC_50_) were 8.5, 2.7, 1.6 and 0.12 μM for *T*. *b*. *brucei* 427 90–13, *T*. *b*. *brucei* Antat 1.1, *T*. *b*. *gambiense* Feo (Feo/ITMAP/1893) and *T*. *b*. *gambiense* Biyamina (MHOM/SD/82), respectively. During infection with these last three virulent strains, posaconazole-eflornithine and nifurtimox-eflornithine combinations showed similar improvement in mice survival (*p*≤0.001). Our results provide support for a CYP51 targeting based treatment in HAT. Thus posaconazole used in combination may represent a therapeutic alternative for trypanosomiasis.

## Author Summary

Human African trypanosomiasis (HAT), also known as sleeping sickness, is a parasitic disease exclusively located in sub-Saharan and southern Africa where it is transmitted by infected tsetse flies and is caused by a blood parasite named *Trypanosoma brucei*. Because of limited alternatives, toxicity and high expense new treatment options are urgently needed for patients with this deadly disease and related diseases. Sterols are important for building cell membranes and are precursors for the synthesis of powerful bioactive molecules, such as steroid hormones in mammals. Among the enzymes required for sterol production, one class (named CYP51), share similar functions and importantly these enzymes are found in all biological kingdoms, including in the sleeping sickness parasite. We have studied the role of trypanosome CYP51 and found that when native levels are diminished experimentally, parasite survival and division are radically diminished resulting in cell death but this can be rescued by the addition of ergosterol. We observed that parasites with diminished level of CYP51 enzyme were less virulent in mice. We also show that posaconazole, a clinically used antifungal and potential CYP51 inhibitor, augments parasite killing when used in combination with currently used trypanocidal drugs. Thus our results suggest that a specific targeting of the enzyme could be a potential strategy to treat HAT.

## Introduction

*Trypanosoma brucei gambiense* and *rhodesiense* are the causative agents of Human African Trypanosomiasis (HAT), also known as sleeping sickness. These flagellated protozoan parasites live and multiply extracellularly in the blood and tissue fluids of the host. *Trypanosoma brucei gambiense* causes a chronic form of HAT in West and Central Africa that progress in two stages: a first early or hemolymphatic stage, and a second late or meningoencephalitic stage [1]. Treatment for first-stage relies on pentamidine and treatment for second-stage relies on nifurtimox + eflornithine (α-difluoromethylornithine, DFMO) combination therapy (NECT) since 2009 [2,3]. Eflornithine is an inhibitor of the first step of polyamine biosynthesis, the formation of putrescine from ornithine by ornithine decarboxylase, and was registered more than 25 years ago in HAT treatment [4,5]. Attempts to reduce eflornithine-based therapy have resulted in its evaluation in combination with nifurtimox [6]. Nifurtimox is believed to exert its biological activity through superoxide anions and nitro anion radicals generated following reduction of the nitro-group, which is attached to the aromatic ring [7]. Nevertheless, nifurtimox has many adverse effects such as: gastrointestinal manifestations, weight loss, fatigue, headache, mood changes, arthralgia, myalgia, skin rash and peripheral neuropathy [8]. It is also considered as genotoxic. Thus, safer and effective therapeutic options for patients with HAT are clearly needed.

Sterol biosynthesis is a crucial pathway in eukaryotes leading to the production of cholesterol in animals and various C24-alkyl sterols (ergostane-based sterols) in fungi, plants, and *Trypanosomatidae*. Sterols are important membrane components and precursors for the synthesis of bioactive molecules, including steroid hormones in mammals. Precise functions of sterol synthesis in protozoa, however, are not well characterized. In these parasites, sterols are produced from acetyl-CoA *via* a multistep metabolic pathway. Sterol 14alpha-demethylase (cytochrome P450 family 51 (CYP51)) catalyzes removal of a 14alpha-methyl group from lanosterol [9,10]. Recently, CYP51 has been shown as essential in *Leishmania donovani* [11]. Nevertheless, data are limited regarding *T*. *brucei* sterol biosynthetic pathway. Contrary to other *Trypanosomatidae*, *T*. *brucei* bloodstream forms are known to build their membranes using host cholesterol from the human plasma [12]. However, it has been shown that, under conditions of cholesterol abundance, parasite endogenous sterol biosynthesis is downregulated but not eliminated completely [13,14]. The very small amount of ergosterol derivatives in *T*. *brucei* suggests that, in the mammalian host, *T*. *brucei* require functional endogenous sterols as metabolic or signal molecules [13,15]. To date, data are nevertheless lacking regarding the essentiality of CYP51 in *T*. *brucei* bloodstream forms [16,17].

It has been previously reported that some azole derivatives are active *in vitro* against *T*. *brucei* bloodstream forms [18]. Alterations in the sterol composition of the parasite are observed upon these treatments, with decrease in the ergosterol formation and accumulation of the C14 methylated precursors providing direct evidence that the mode of action of these drugs is connected with CYP51 inhibition. Posaconazole is a registered extended-spectrum triazole with demonstrated efficacy as antifungal treatment for human refractory invasive fungal infections [19,20] and as prophylaxis in high-risk patients [21,22]. It is usually considered as well-tolerated, although some adverse events have been reported such as diarrhea, headache or fever [23]. Elevation of serum transaminase concentrations is infrequent (2%-3%) [24]. Crystal structures of CYP51 from *T*. *brucei* have been determined and its binding to posaconazole has been studied [25,26]. Additionally, posaconazole was shown to be active in experimental models caused by related parasites such as *Leishmania amazonensis* and *donovani* [27,28] or *Trypanosoma cruzi* [29]. During HAT, combined therapy is envisioned as an approach to favor since it may improve treatment efficacy whilst decreasing toxicity and the likelihood of resistance development. To date, no data on posaconazole effect in HAT are available.

Given the interest of CYP51 as a drug target and the severity of disease caused by *T*. *brucei*, we investigated the essentiality of *T*. *brucei* CYP51 and we evaluated the effect of posaconazole alone and in combination with eflornithine *in vitro* and in a murine model of African trypanosomiasis.

## Material and Methods

### Parasites

For *in vitro* experiments on a model strain, we used the bloodstream form (BSF) *T*. *brucei brucei* 427 90.13 cell line, co-expressing the T7 RNA polymerase and tetracycline repressor, named wild type (WT) in this study [30]. WT parasites were grown in IMDM medium as described in [31] containing 10% heat-inactivated fetal calf serum, 36 mM sodium bicarbonate, 136 μg/ml hypoxanthine, 39 μg/ml thymidine, 110 μg/ml sodium pyruvate, 28 μg/ml bathocuprone, 0.25 mM β-mercaptoethanol, 2 mM L-cysteine, kanamycin (62.5 μg/mL), neomycin (2.5 μg/mL), and hygromycin (5 μg/mL) (from now on called complete IMDM). For animal study, we used the following three pleomorphic strains: *T*. *b*. *brucei* Antat 1.1 (*T*. *b*. *brucei* Antat 1.1 EATRO 1125), *T*. *b*. *gambiense* Biyamina (MHOM/SD/82) and *T*. *b*. *gambiense* Feo (Feo/ITMAP/1893) [32–35]. These last two strains belong to *T*. *b*. *gambiense* group 2. Indeed, *T*. *b*. *gambiense* Biyamina has been previously confirmed as group 2 [36]. We also identified, using mini- and micro-satellite analysis, that *T*. *b*. *gambiense* Feo also belongs to group 2 [37–39] (S1 Table). These pathogenic strains are currently conserved as frozen stabilates in our laboratory. *Leishmania tarentolae* (strain LEXSY host T7-TR, Jena Bioscience GmbH, Germany) was used for western-blot control.

### Construction of plasmid and transfection

We used the tetracycline-inducible RNAi interference (RNAi) system [30,40–42] to assess the essentiality of CYP51 in *T*. *brucei* 427 90.13 BSF cells. The *CYP51* gene (accession number Tb927.11.6210) was identified from the TriTrypDB kinetoplastid genomics resource (http://tritrypdb.org) [43]. A portion (bp 389 to 855) of *CYP51* was amplified by polymerase chain reaction (PCR) from *T*. *brucei* 927 genomic DNA with the primers 899-F (5′-GCCGGCCGCTCTAGATTGCGGAGGAATTAACCATCGC-3′) and 900-R (5′-TAAGCTTGCTCTAGATCATGCCACATACCTCGTGTAGG-3′). The PCR product was purified and the 496 bp fragment was cloned in p2T7tiB plasmid [44] digested with *Xba*I using the In-Fusion cloning kit (Clontech), and the sequence was checked by DNA sequencing. The *Not*I linearized plasmid was transfected by electroporation in *T*. *brucei* 427 90–13 as described in [45]. After 24 hours, transfected cells were diluted in culture medium supplemented with phleomycin (2.5 μg/mL) and selected after cloning by serial dilution into 24-well plates. After 5 to 10 days, transformants were induced with tetracycline (10 μg/mL) and several clones were collected and displayed similar growth reduction upon induction. One clone was selected for further studies. To verify for ergosterol biosynthesis specificity, the rescue of the CYP51^*RNAi*^phenotype was tested by adding 5 μM ergosterol (Sigma catalogue # E6510) in the culture medium.

### Western blotting

In order to study CYP51 expression, cells extracts from the different strains (2.5 x 10^6^ cells, virulent *T*. *brucei* and *T*. *b*. *gambiense* purified from rodent blood, *T*. *brucei* 427 90–13 WT, CYP51^*RNAi*^ non-induced and CYP51^*RNAi*^ induced) were loaded on a 10% SDS-PAGE and transferred onto PVDF membrane. Membranes were blocked in blocking solution (tris-buffered saline (TBS), 0.2% Tween-20, 5% skimmed milk powder) for 1 hour, and incubated overnight at 4°C with the primary antibody anti-CYP51 (A kind gift from Dr. James. H. McKerrow, Skaggs School of Pharmacy and Pharmaceutical Sciences, University of California San Diego, U.S.A) [11] diluted in blocking solution at 1:5,000. After 3 washes (10 min) in TBS, 0.2% Tween-20, the membranes were incubated for 1 hour at room temperature with anti-rabbit HRP (horseradish peroxidase)-conjugated secondary antibody (Sigma A-9169) diluted at 1:10,000 in blocking solution. After 3 washes in blocking solution and 2 washes in TBS, membranes were revealed by ECL (Clarity Biorad chemiluminescence). Membranes were stripped 2 times 5 min in glycine 100 mM pH2.3, 1% SDS, 0.1% NP40 (Igepal), washed in TBS for 30 min, blocked in blocking solution for 1hr and processed with the loading control anti-GAPDH (a kind gift from Paul Michels, University of Edinburgh, U.K.) at 1:1,000 dilution. The signals of three independent experiments were quantified using ChemiDoc XRS+ (Biorad) and Image Lab 5.1 software.

### Immunofluorescence

WT, CYP51^*RNAi*^ non-induced and induced cells were fixed in 3% paraformaldehyde in PBS for 2 minutes. After PFA fixation (3%), glycine was added (100 mM in PBS, 10 min) and cells were spread on poly-L-lysine-coated slides. Cells were permeabilized with Triton-X100 0.2% in PBS for 10 min and washed once in PBS. Samples were incubated with the primary antibody for 1 hour at room temperature in a moist chamber: anti-PFR (mouse monoclonal L8C4, neat, a kind gift from Pr. K. Gull, Oxford University). After two PBS washes, cells were incubated for 45 min with the secondary antibody anti-mouse conjugated to FITC (Sigma F-2012, 1:100 dilution). Nuclei and kinetoplasts were labeled with DAPI (10 μg.mL^-1^ in PBS for 5 minutes) and washed twice in PBS for 5 minutes. Slides were mounted with Slowfade Gold (Molecular Probes S-36936).

### Effect of posaconazole on CYP51^*RNAi*^ cells

Cultures were performed in 24-well plates. Each well was filled with 500 μL of complete IMDM medium. Posaconazole was dissolved in dimethyl sulfoxide (DMSO) (6 mM stock) and stored at -80°C. For experiments, new dilutions were prepared in culture medium to ensure that the DMSO concentration in the culture medium did not exceed 0.1%. Adequate dilutions of the drug were added to 2 x 10^5^/mL WT, non-induced and RNAi induced parasites with tetracycline (10 μg/mL), after 5 days and 10 days of RNAi induction. Cultures were maintained at 37°C in 5% CO_2_ incubator for 24 hours. Parasite counts were then done in a Malassez. Results were expressed as dose-effect curves. Growth curve of WT parasites incubated with DMSO 0.1% were similar to control. Experiments were performed three independent times and mean and standard error to the mean (sem) were calculated.

### Effect of posaconazole *in vitro* on animal strains

*T*. *b*. *brucei* Antat 1.1, *T*. *b*. *gambiense* Biyamina or *T*. *b*. *gambiense* Feo stabilates were de-frosted, inoculated into mice and then purified from rodent blood using DEAE-cellulose columns, as previously described [46]. Assays were performed in 96-well plates. Each well was filled with 10^5^ parasites in 200 μL of RPMI culture medium, with appropriate dilutions of drugs, supplemented with 10% FCS, 100 U/mL penicillin, 100 μg/mL streptomycin, 25 mM HEPES, 2 mM sodium pyruvate and 0.1 mM 2-mercaptoethanol (from now on called complete RPMI) [32,47]. Cultures were maintained at 37°C in 5% CO_2_ incubator for 24 hours and the efficacy was expressed in concentration inhibiting parasite growth by 50% (IC_50_) [48]. Experiments were performed three independent times and the mean and sem of the IC_50_ for each drug were calculated.

Additionally, in order to test if posaconazole effect is dependent on the sterol biosynthesis pathway in pleomorphic strains, we cultured bloodstream *T*. *b*. *brucei* Antat 1.1 parasites in the presence of ergosterol and different amounts of posaconazole. Ergosterol (Sigma catalogue # E6510) was dissolved in chloroform as 125 mM stock. For experiments, ergosterol was diluted at 50 μM concentration as used in [49]. Dilutions were prepared to ensure that the chloroform concentration in the culture medium did not exceed 0.01%. Ergosterol, posaconazole or their combinations were added at the same time to cultures.

### Animal model

This investigation conformed to the Guide for the Care and Use of Laboratory Animals (NIH Publication No. 85–23, revised 1996). Agreement (number A33-063-324) was obtained from French authorities and all the protocols used were approved by our local ethics committee *Comité d'éthique régional d'Aquitaine* (protocol number: 215122414261294). Female Swiss (OF-1) mice eight to ten weeks old, 18–25 g, (Charles River, L’Arbresle, France) were kept in our animal housing facility fifteen days before the experiment was started. They were housed six mice to a cage in ventilated boxes kept in a protected temperature and humidity-controlled room, with a 12 hours on/off light cycle. The animals were given free access to food and water. Efforts were made to minimize the suffering of animals used.

In order to test the effect of CYP51 RNAi on parasite virulence, mice (eight per group) were infected at day 1 by subcutaneous injection with 10^4^ WT, CYP51^*RNAi*^ non-induced or CYP51^*RNAi*^ pre-induced (*in vitro* for 5 days with tetracycline) parasites. Mice were given either normal water or water containing 200 μg/mL doxycycline in a 5% sucrose solution [50]. The drinking water with or without doxycycline was provided 3 days before infection and changed every day. Parasitemia levels were monitored every day during the whole experiment.

In order to test the effect of the different drugs used, mice were infected at day 1 by subcutaneous injection with 10^4^ bloodstream form parasites of *T*. *b*. *brucei* Antat 1.1 (twelve mice per group), *T*. *b*. *gambiense* Biyamina (MHOM/SD/82) (twelve mice per group) or *T*. *b*. *gambiense* Feo (Feo/ITMAP/1893) (eight mice per group). After inoculation, mice were randomly separated into four groups of treatment for each strain. The different groups were: (i) controls, (ii) posaconazole, (iii) eflornithine, (iv) posaconazole + eflornithine and (v) nifurtimox + eflornithine. Treatments started at day five post-inoculation. In order to mimic the NECT treatment, eflornithine was given the first seven days of treatment, while nifurtimox was given ten days. Nifurtimox was administered at a total of 20 mg/kg/day and was given in two doses by oral gavage with 12 hours between each dose. Eflornithine was continuously available in the drinking water, over seven consecutive days. Each mouse consumed an average of 3 mL of 2% eflornithine per day, which yielded a mean dosage of 3.2 g/kg of body weight per day [4,51]. Posaconazole was provided as Noxafil oral suspension (Merck Sharp & Dohme Corp.) and was administered twice a day by oral gavage. For the posaconazole + eflornithine combination, eflornithine was given the first seven days of therapy (from day 5 to day 11 post-inoculation) and posaconazole was given for ten days (from day 5 to day 14 post-inoculation). Blood samples were taken daily from the tip of the tail to assess parasitemia [52]. Animals were followed for survival or relapse until day 180.

### Data analysis

Data were expressed as mean ± standard error to the mean (sem) of n experiments. *In vitro* data were analyzed using a Mann-Whitney non-parametric test. Animal survival was analyzed using Kaplan-Meier survival plots and log rank test. Statistical analyses were done using STATA software (version 9.2, Stata corporation, college station, TX). Values were considered statistically significant with *p*<0.05.

## Results

### *Tb*CYP51 gene is conserved and syntenic

*T*. *b*. *gambiense* Biyamina (MHOM/SD/82) and *T*. *b*. *gambiense* Feo (Feo/ITMAP/1893) were used in these studies and these strains belong to *T*. *b*. *gambiense* group 2 (S1 Table). A single-copy gene, located on chromosome 11 of the *T*. *brucei brucei* genome, encodes the *Tb*CYP51 (sterol 14-alpha-demethylsase) protein (Tb927.11.6210). BLAST analysis, using parasite TriTrypB database, identified an 84% sequence identity between *T*. *brucei and Leishmania donovani* (*Ld*). Conversely, the amino acid sequence identity to mammalian CYP51 was low (24%). CYP51 genes shown very similar locations with regard to their respective flanking genes amongst *Trypanosomatidae* (*T*. *brucei gambiense*, *T*. *congolense*, *T*. *vivax*, *T*. *cruzi*, *Leishmania major* and *L*. *donovani)*, indicating that *CYP51* gene synteny is preserved amongst these species.

### *Tb*CYP51 is expressed in bloodstream form

**Fig 1:**
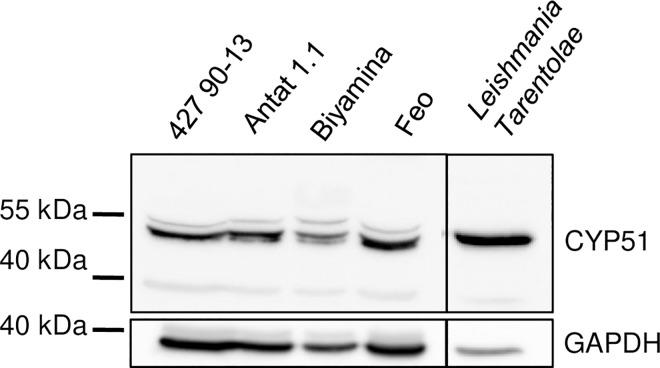
*Tb*CYP51 is expressed in *T*. *brucei* bloodstream forms. Western blot analysis of *Tb*CYP51 expression in bloodstream forms of *T*. *b*. *brucei* Antat 1.1, *T*. *b*. *gambiense* Biyamina, *T*. *b*. *gambiense* Feo and *Leishmania tarentolae* strains using anti-*Ld*CYP51. Lower panel is anti-GAPDH loading control.

*TbCYP51* encodes a typical CYP51 protein with a predicted signal peptide at the N-terminus (aa 1–24). *Tb*CYP51 is a 481 aa protein with a predicted molecular mass of 51.5 kDa after signal peptide cleavage. *Tb*CYP51 has been the subject of structural studies [26,53,54] but, to our knowledge, there was no direct evidence of expression of this protein in *T*. *brucei* BSF. As *Tb*CYP51 and *Ld*CYP51 proteins share 84% of identity, we used the anti-*Ld*CYP51 antibody described by McCall and colleagues [11] to probe the kinetoplastids used in this study by western-blot. A band at the expected size was observed demonstrating that *Tb*CYP51 is expressed in *T*. *brucei* BSF and *T*. *b*.*gambiense* cells (Fig 1).

### Ergosterol can rescue lethal CYP51^*RNAi*^ knockdown phenotypes in bloodstream forms

**Fig 2:**
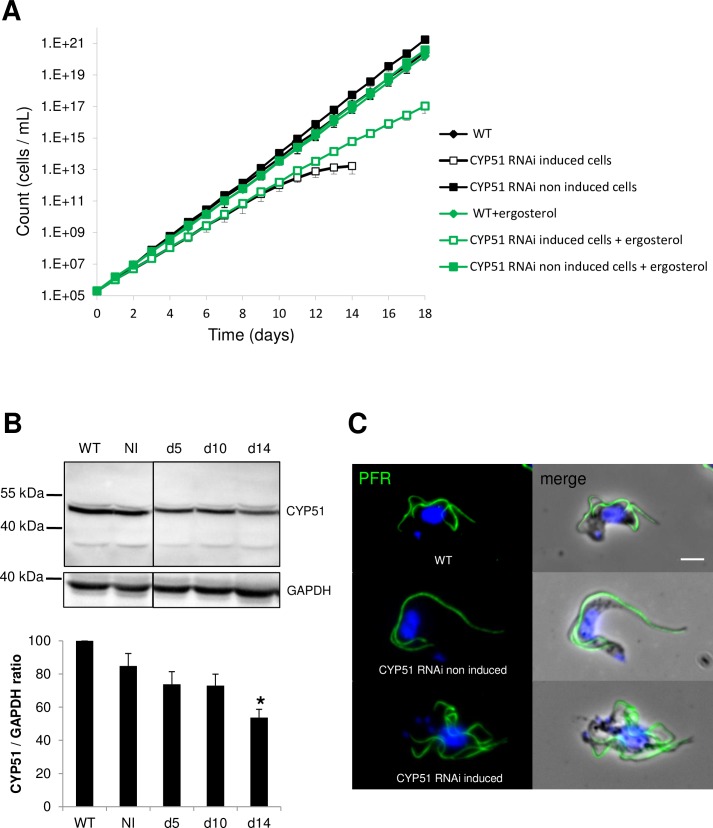
*Tb*CYP51 RNAi knockdown is lethal in BSF and can be rescued by ergosterol. A. Growth curve of WT, non-induced and *Tb*CYP51^*RNAi*^ induced cells plus or minus ergosterol (5 μM) indicating cell growth arrest and death after 14 days of induction but rescue in the presence of ergosterol. B. Upper panel: Western blot of CYP51 for Wild Type (WT), non-induced (NI) and *Tb*CYP51^*RNAi*^ induced cells at day 5 (d5), day 10 (d10) or day 14 (d14). Lower panel: quantification of the western blot. *p<0.05. C. Phenotype analysis by immunofluorescence using anti-PFR2 antibody (green). Nuclei and kinetoplasts are DAPI stained (blue). Scale bar represents 5 μm. Error bars in A, B represent the standard error to the mean (sem) from 3 independent experiments.

To assess the functional role of *Tb*CYP51 in the parasite, we used the tetracycline-inducible RNA interference (RNAi) system in *T*. *brucei* 427 90–13 BSF cells [30]. From day 1, a reduced growth rate was observed for induced cells compared to the non-transformed parental cell line (WT) and the non-induced CYP51^*RNAi*^ cells. Cell growth arrest and cell death were observed after 14 days of induction. Addition of 5 μM ergosterol to the culture medium rescued the growth defect phenotypes, demonstrating that *Tb*CYP51 is essential in *T*. *brucei* BSF (Fig 2A). Western-blot analysis of *Tb*CYP51 expression level showed that RNAi induction reduced the expression of the protein (Fig 2B upper panel). Indeed, densitometry data indicate that at 5 days of induction, CYP51 protein levels had lowered by 26.0% (sem = 7.5) compared to WT levels and by 27.0% (sem = 6.9) and by 46.0% (sem = 5.8) at 10 days and 14 days (*p*<0.05), respectively (Fig 2B lower panel). A slight reduction of expression (18%, sem = 8.0) was also observed in non-induced cells when compared to WT cells, but this suggests that a slight decrease in CYP51 expression does not affect cell growth. Taken all together, these results suggest that ergosterol synthesis *viaTb*CYP51 is essential in *T*. *brucei* BSF.

CYP51^*RNAi*^ induction also induced some morphological defects. By immuno-fluorescence labeling using an antibody directed to the paraflagellar rod (a structure of the flagellum involved in motility) [55], we observed that CYP51^*RNAi*^ induction led to multiflagellated cells, a landmark for cytokinesis defect in BSF (Fig 2C). Mean multiflagellated cell counts in three experiments were: 5.8% (sem = 0.6), 8.7% (sem = 2.6) and 53.7% (sem = 5.0) in WT, non-induced and 5 days induced cells, respectively (*p*<0.05). At d14 of induction, PFR labelling was of poor quality because most of the cells were dead or dying, and therefore could not be clearly counted, however multiflagellated cells were still observable.

### RNAi knockdown of *Tb*CYP51 reduces sensitivity to posaconazole

**Fig 3:**
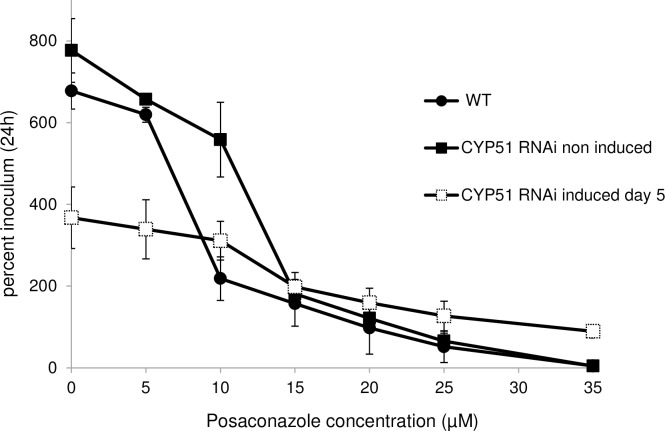
Induced-*Tb*CYP51^*RNAi*^ cells are less sensitive to posaconazole. Dose-effect curves of posaconazole for WT, non-induced and CYP51^*RNAi*^ induced cells at day 5 (d5). Cells were inoculated at 2 x 10^5^cells/mL and incubated in complete IMDM medium supplemented with 10% FCS and with increasing concentrations of posaconazole (from 0 to 35 μM). After 24 hours of treatment, cell number was calculated and expressed graphically as a percentage of the initial inoculum. Error bars represent the standard error to the mean (sem) from 5 independent experiments.

Adequate dilutions of posaconazole were added to 2 x 10^5^ WT parasites to determine the concentration inhibiting parasite growth by 50% (IC_50_). Dose-effect curves at 24 hours of culture showed that the WT strain was sensitive to posaconazole with an IC_50_ of 10 μM. This posaconazole dose-effect curve was repeated (n = 5) with non-induced and CYP51^*RNAi*^ induced cells to assess the posaconazole sensitivity when CYP51 expression is reduced. After 5 days of CYP51^*RNAi*^ induction, cells were incubated with increasing concentrations of posaconazole for 24 hours, and the parasites were counted (Fig 3). Interestingly, CYP51^*RNAi*^ induced cells were less sensitive to posaconazole than WT cells (IC_50_ was 8.5 μM) since the IC_50_ of non-induced and CYP51^*RNAi*^ induced cells were 12.3 μM and 16.5 μM respectively, reflecting the reduction of *Tb*CYP51 expression in non-induced and in induced cells as previously observed by western-blot. This suggests a functional link between CYP51 and posaconazole mechanism of action.

### RNAi knockdown of *Tb*CYP51 reduces virulence in a mouse model

**Fig 4:**
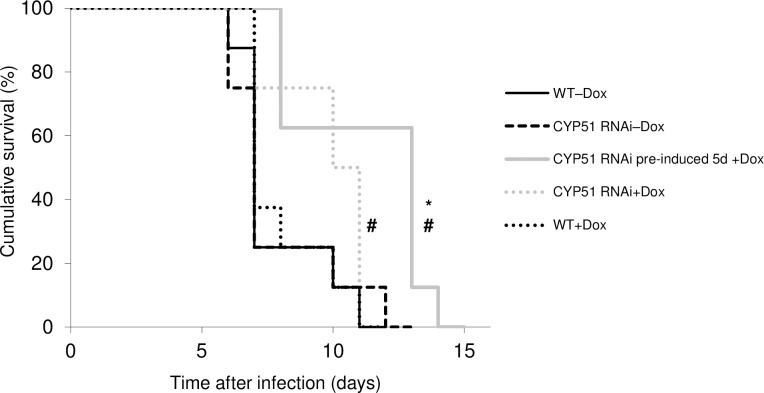
The effect of CYP51 RNAi on parasite virulence. In order to test the effect of CYP51 RNAi on parasite virulence, mice were infected at day 1 by subcutaneous injection with 10^4^ WT, or non-induced CYP51^*RNAi*^, or with CYP51^*RNAi*^ pre-induced (5 days) parasites. Mice (8 per group), were given either untreated water or water containing 200μg/mL Doxycycline in a 5% sucrose solution. The drinking water with or without Doxycycline was provided 3 days before infection and changed daily. The survival of untreated Doxycycline WT (WT–Dox), Doxycycline-treated WT (WT+Dox), and CYP51^*RNAi*^ Doxycycline-untreated (CYP51^*RNAi*^–Dox) groups of infected mice were compared, by pairs, using survival analysis test (Kaplan-Meier and log rank test. ^#^Significant *p*<0.05 compared to CYP51^*RNAi*^–Dox (non-induced cell). *Significant *p*<0.05 compared to CYP51^*RNAi*^–Dox, WT–Dox and WT+Dox.

In order to test the role of *Tb*CYP51 in virulence and *in vivo*, we infected doxycycline treated and untreated mice with CYP51^RNAi^ parasites or with CYP51^RNAi^ parasites that were pre-induced in culture for 5 days (Fig 4), and then monitored mouse survival rates. Parasites were pre-induced for five days because the WT and the non-induced CYP51^*RNAi*^ strains caused an acute infection in mice, which killed them on average at day 7 *i*.*e*., before RNAi-induced cell death observed *in vitro*. The survival of Doxycycline un-treated WT (WT–Dox), Doxycycline-treated WT (WT+Dox), and CYP51^*RNAi*^ Doxycycline-untreated (CYP51^*RNAi*^–Dox) groups of infected mice were compared, by pairs, using survival analysis test (Kaplan-Meier and log rank test), and showed no difference since the Log rank *p* values were comprised between 0.74 and 0.79. The CYP51^*RNAi*^ Doxycycline-treated group (CYP51^*RNAi*^+Dox) showed improved survival compared to CYP51^*RNAi*^–Dox (*p* = 0.044), and to WT–Dox or WT+Dox (*p* = 0.053 and *p* = 0.062 respectively). Further, the 5-day pre-induced Doxycycline-treated group (CYP51^*RNAi*^ pre-induced 5d +Dox) showed significant improved survival compared to CYP51^*RNAi*^–Dox, and to WT–Dox or WT+Dox (*p* = 0.007, *p* = 0.008, *p* = 0.013 respectively). This shows that knockdown of expression of *Tb*CYP51 *in vivo* reduces parasite virulence.

### Posaconazole is effective *in vitro* on animal strains

The long-term objective of this study is to target *Tb*CYP51 *in vivo*. We thus first assess the sensitivity to posaconazole, nifurtimox, eflornithine, and pentamidine of the infectious strains *T*. *b*. *brucei* Antat 1.1, *T*. *b*. *gambiense* Biyamina, and *T*. *b*. *gambiense* Feo [32–35]. These strains were used to inoculate mice, parasitemia was checked, and parasites were purified from blood on DEAE-cellulose columns. Parasites were then incubated for 24 hours in medium containing different concentrations of the above mentioned compounds. The IC_50_ results are reported in Table 1. The cytotoxic activities were similar between posaconazole and nifurtimox, for the same strain. Combination testing with determination of the Fractional Inhibitory Concentration (FIC) Index was performed for different drug combinations [56]. We checked for synergistic effects when nifurtimox plus eflornithine (NECT) or posaconazole plus eflornithine (PECT) or posaconazole plus nifurtimox (PON) when tested in the pairs as mentioned *i*.*e*., NECT, PECT or PON, or alone. Although a pair was more effective than individual drugs, we did not find any synergistic nor antagonistic effect. All the combinations of drugs were additive or indifferent, with fractional inhibitory concentration index ≥1.0 and ≤4.0.

**Table 1:** *In vitro* killing activity of selected drugs on animal virulent strains (IC_50_). Each strain was incubated in complete RPMI culture medium containing different concentration of drugs. The concentration that inhibited parasite growth by 50% (IC_50_ μM) was determined after 24 hours of incubation.

	*T*. *b*. *brucei* Antat 1.1	*T*. *b*. *gambiense* Biyamina	*T*. *b*. *gambiense* Feo
Posaconazole	2.7 ± 1.1	0.12 ± 0.01	1.6 ± 0.2
Nifurtimox	1.7 ± 0.5	0.08 ± 0.01	0.8 ± 0.1
Eflornithine	33.3 ±7.9	25.0 ±2.6	20.8 ±2.2
Pentamidine	0.6 ± 0.2	0.4 ± 0.06	0.05 ± 0.01

Each result is the mean ± sem, from 3 independent experiments

**Fig 5:**
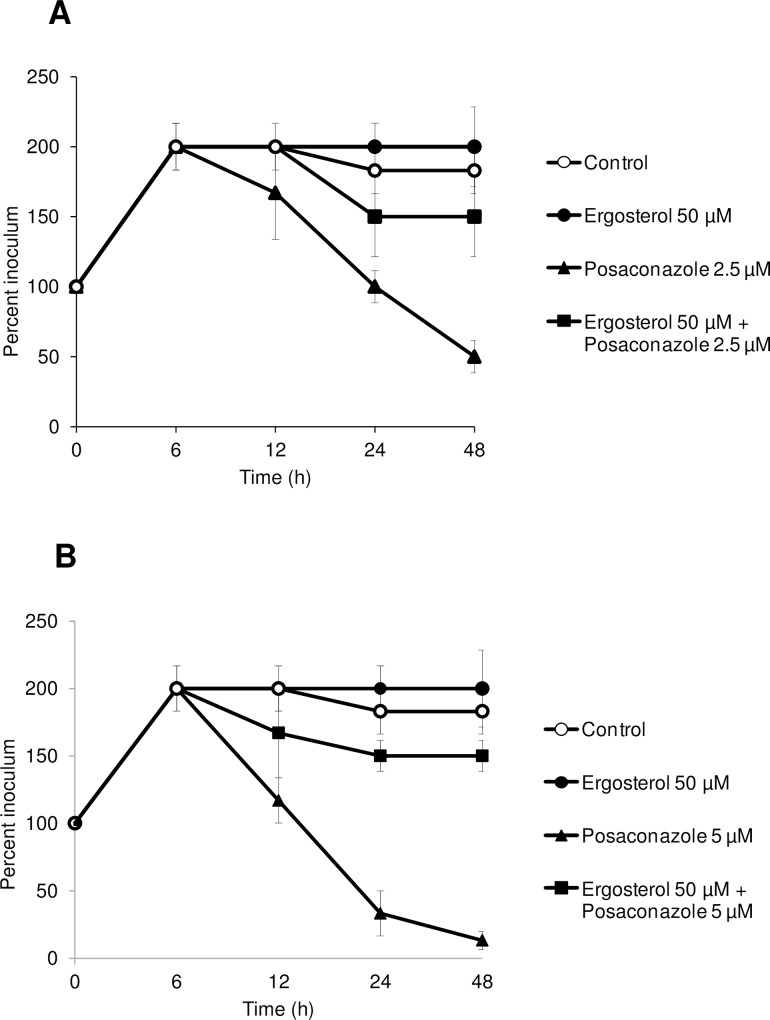
Reversing the effect of posaconazole using ergosterol. Posaconazole was tested at 2.5 μM (A) and 5 μM (B). *T*. *brucei* Antat 1.1 BSF parasites were inoculated at 5.0 x 10^5^ cells/mL and incubated in complete RPMI medium in absence or in presence of posaconazole at 2.5 μM (A) or 5 μM (B), ergosterol (50 μM), or a combination or ergosterol 50 μM + posaconazole (2.5 μM in A, 5 μM in B) for 48 hours. Cell survival was assessed at 6, 12, 24, 48 hours of incubation and cell number was calculated and expressed graphically as a percentage of the initial inoculum. Error bars represent the standard error to the mean (sem) from 4 independent experiments.

Additionally, to confirm that the posaconazole effect is indeed dependent on the ergosterol biosynthesis pathway in animal pathogenic parasites, we cultured bloodstream *T*. *b*. *brucei* Antat 1.1 parasites in presence of ergosterol plus different concentrations of posaconazole. It was observed that ergosterol reversed the effect of posaconazole, when using posaconazole at the IC_50_ (2.5 μM) and IC_90_ (5 μM) concentrations (Fig 5A and 5B).

### The posaconazole-eflornithine combination (PECT) is an alternative to NECT in a mouse model

A preliminary experiment with the *T*. *b*. *brucei* Antat 1.1 strain, with increasing doses of posaconazole, (5, 10, 20 or 50 mg/kg/day given in two doses and with 12 hours between each dose), was performed to determine an effective dose. All mice developed an infection and those that died had high parasitemia (>10^6^/mL). The mice treated with 20 mg/kg/day twice a day had significantly prolonged survival than controls (*p* = 0.008). This latter dose was retained as effective and used for the following experiments.

**Fig 6:**
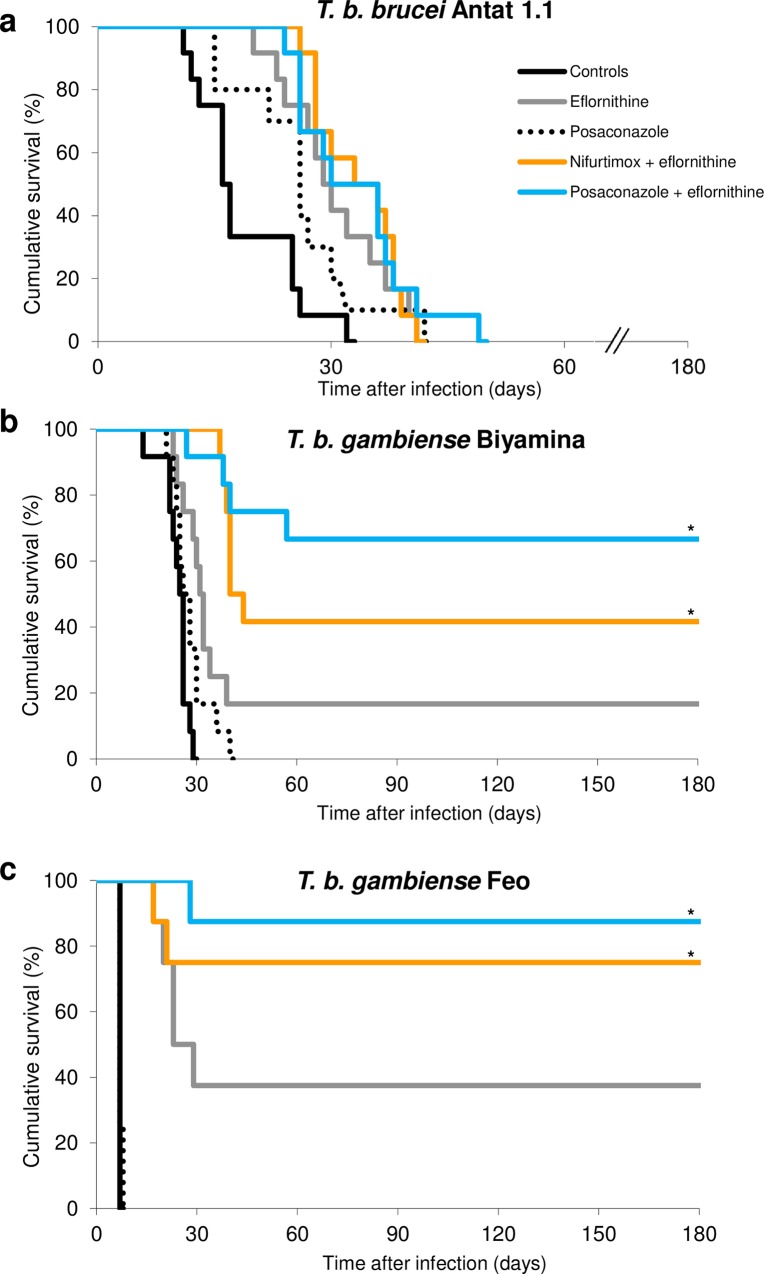
The posaconazole-eflornithine combination (PECT) is an alternative to NECT in a mouse model. Survival curves using the combinations of drugs for mice infected with *T*. *b*. *brucei* Antat 1.1 (Fig 6a), *T*. *b*. *gambiense* Biyamina (Fig 6b) and *T*. *b*. *gambiense* Feo (Fig 6c). Treatments were started at day five post-inoculation. Nifurtimox and posaconazole were administered at 20 mg/kg/day twice a day by oral gavage and eflornithine at 3.2 g/kg in the drinking water. Animals were followed for survival or relapse until day 180. The *T*. *b*. *brucei* Antat 1.1, PECT and NECT groups showed improved survival compared to controls (*p* = 0.001). With regards to *T*. *b*. *gambiense* Biyamina, the PECT and NECT groups showed improved survival compared to controls (*p*<10^−4^) and to eflornithine group (*p* = 0.003 and *p* = 0.01, respectively). *T*. *b*. *gambiense Feo*, the PECT and NECT groups showed improved survival compared to controls (*p*<10^−4^) and to eflornithine group (*p* = 0.02 and *p* = 0.03, respectively). *Significant *p*<0.05 compared to controls and to eflornithine group.

We next determined the survival rates of mice infected with *T*. *b*. *brucei* Antat 1.1 (Fig 6A), *T*. *b*. *gambiense* Biyamina (Fig 6B) and *T*. *b*. *gambiense* Feo (Fig 6C), and subjected them to NECT treatment (nifurtimox+eflornithine), eflornithine, posaconazole, and PECT treatment (posaconazole+eflornithine). For each of these three experiments, the over-all log rank test showed a difference between the set of curves (*p*<10^−4^). *T*. *b*. *brucei* Antat 1.1 mice treated with posaconazole or mice treated with eflornithine showed improved survival compared to controls (n = 12 in each group; log rank *p* = 0.008 and *p* = 0.001, respectively). The PECT and NECT groups showed improved survival compared to controls (*p* = 0.001) but not compared to eflornithine group (*p* = 0.4). There was no significant difference between the survival with PECT and NECT (*p* = 0.97). With regards to *T*. *b*. *gambiense* Biyamina, the PECT and NECT groups showed improved survival compared to the posaconazole treated group and to controls (n = 12 in each group; log rank: *p*<10^−4^). Moreover, the PECT and NECT groups showed improved survival compared to eflornithine group (*p* = 0.003 and *p* = 0.01, respectively). The survival was globally better with PECT than with NECT, with eight out of twelve, and five out of twelve mice cured, respectively. Nevertheless, the difference between PECT and NECT groups was not statistically significant (log rank: *p* = 0.14). Pertaining to *T*. *b*. *gambiense Feo*, the PECT and NECT groups showed improved survival compared to the posaconazole treated group and to controls (n = 8 in each group; log rank: *p*<10^−4^). PECT and NECT groups showed improved survival compared to eflornithine group (*p* = 0.02 and *p* = 0.03, respectively). Seven out of eight, and six out of eight mice were cured with PECT and NECT combinations respectively, without any significant statistical difference (log rank: *p* = 0.48).

## Discussion

Our study demonstrates the essentiality of CYP51 in *T*. *brucei*, using a tetracycline-inducible RNAi system. We observed morphological defects with multiflagellated cells, and cytokinesis dysfunction, which are phenotypes often observed when the function of important proteins is perturbed or knocked down in bloodstream forms. Moreover, by testing the effects of posaconazole on CYP51^*RNAi*^ cells and on virulent animal strains, we observed that this drug has an anti-trypanosomal activity, probably connected with a mechanism of action targeting CYP51. Importantly, the effect of posaconazole at 20 mg/kg combined with eflornithine (PECT) was similar to NECT combination in infected-mice.

CYP51 enzymes are found in all biological kingdoms and have been joined into one cytochrome P450 family (CYP51) because of their strict functional conservation, despite their low amino acid sequence identity, ranging from 22 to 27% across phylogeny [57]. Among the *Trypanosomatidae*, the essentiality of CYP51 has been demonstrated for *Leishmania donovani* [11] and *Leishmania major* [58]. In the latter study, CYP51-null mutants were viable but show defects in growth rate, cytokinesis defects and hypersensitivity to heat stress. We observed that 53.7% of 5-days CYP51^*RNAi*^ induced *T*. *brucei* were multiflagellated, whereas only 5.8% of parental parasites presented this abnormality. Moreover, in our work, cell growth was reduced in CYP51^*RNAi*^ induced cells compared to WT with cell growth arrest after 12 days, followed by death after 14 days. These observations are in line with the progressive slow decrease in CYP51 expression that we measured by Western blot and that could be explained by a long half-life of this protein. Cell death occurred in our study when CYP51 expression level approaches 50% of the value of WT cells. These results differ from those of Mc Call *et al*. in 2015 who found that *L*. *donovani* parasites were able to tolerate > 50% reduction in CYP51 protein levels with no apparent effects on parasite phenotype [11]. These authors finally confirmed essentiality of CYP51 in *Leishmania* by obtaining complete loss of chromosomal *CYP51* genes only in the presence of an episomal source of CYP51.

We endeavored, without success, to identify the sub-cellular localization of the protein, using *in situ* tagging of CYP51 and expressing this in trypanosomes as described in [59]. Interestingly, the addition of the tag was probably lethal because no transformed clones were obtained despite repeated transformations, and all transfected cultures died. Therefore, our results show that CYP51 is indispensable in *T*. *brucei* bloodstream form. Knockdown of CYP51 induced the common and “archetypal” secondary or downstream effects observed in bloodstream forms including modulation of cell the cycle and cell division.

It is rather surprising that CYP51^*RNAi*^ induced cells are less sensitive to posaconazole than WT cells. Indeed, logic would suggest that when the level of CYP51 is reduced during RNAi induction, the remaining proteins should be targeted by posaconazole, resulting in more rapid death. Interestingly, it has been previously reported quite similar results with CYP51-null mutants of *Leishmania major*, which show increased resistance to itraconazole and amphotericin B [58]. Some hypotheses could explain our observations. First, *T*. *brucei* might be less sensitive because there is little or no target for the drug to bind and block, which could limit the effect of the drug. Secondly, the morphologic changes in cell shape after knockdown could be associated with a diminution in drug uptake. Thirdly, knockdown of CYP51 may induce the accumulation of compensatory substrates or enzymes whose activity prevent or reduce the effect of posaconazole. For instance, it has been hypothesized that, during CYP51 knockdown in *Leishmania*, 14-methylfecosterol and 14-methylzymosterol could partially compensate the loss of ergostane-based sterol and that other membrane lipids (sphingolipids, glycerophospholipids, and cholesterol) may also help stabilize the plasma membrane [58]. A combination of more than one of the above mentioned hypotheses could also influence drug efficacy in induced RNAi cells.

We aimed to evaluate the reversibility of the effect of CYP51 RNAi and of posaconazole on trypanosomes cell growth and cell survival using ergosterol, as previously proposed by some authors in fungi [49]. We observed reversibility of phenotype in CYP51^*RNAi*^ knockdown cells treated with ergosterol, confirming that the mechanism of action of posaconazole is dependent on the sterol biosynthesis pathway in trypanosomes. This result is strengthened by our data showing that CYP51^*RNAi*^ in mice reduces parasite virulence. Whereas the RNAi study tends to validate CYP51 as the target for posaconazole, we cannot, however, exclude a pleiotropic effect on this pathway. Therefore, further studies need to be performed to evaluate the sterol composition of CYP51^*RNAi*^ induced *T*. *brucei* cells or their infectivity in mice.

Different approaches have been previously attempted to target sterol pathway in trypanosomes. Inhibitors of squalene synthase have shown anti-trypanosomal activity against *T*. *b*. *rhodesiense* bloodstream forms *in vitro* [60]. Different enzyme inhibitors targeting CYP51 and C24-methyltransferase have also been identified [18,61–63]. Posaconazole is a potent inhibitor of the Cyp450-dependent lanosterol 14α-demethylase in yeasts and molds [64,65]. It was shown to be active against *Leishmania* [27,28] or *Trypanosoma cruzi* [29] in murine hosts. Due to the limits of *in vitro T*. *gambiense* cultures, our approach has been to complete *in vitro* drug testing by a mouse model. In mammals, numerous factors interact such as immune characteristics of the host, drugs pharmacokinetics, disease stage and timing of treatment. Various models have been proposed to study acute or late trypanosome infections [4,34,35,66,67]. For our studies, we used a panel of three well characterized strains. In our model, *T*. *b*. *gambiense* Feo induces an acute form of trypanosomiasis, whereas *T*. *b*. *gambiense* Biyamina and *T*. *b*. *brucei* Antat 1.1 induce infection evolving during 3 to 4 weeks followed by animal death. *T*. *b*. *brucei* Antat 1.1 penetration into the brain parenchyma have been extensively studied [34,35]. Nevertheless, both the *T*. *b*. Biyamina and *T*. *b*. Feo strains belong to group 2 *gambiense*, which can be seen as a limitation if potential reservoirs are not considered. In our study, when used alone, posaconazole effect was significant, although limited, in prolonging animal survival (*p* = 0.008). When combining with eflornithine, cure rates were close to those of NECT treatment and were variable depending on the strain (0.0%, 68.0% and 88.0% for *T*. *b*. *brucei* Antat 1.1, *T*. *b*. Biyamina and *T*. *b*. Feo, respectively). Of note, mice infected with the *T*. *brucei* Antat 1.1 strain died faster, which was correlated with higher IC_50_ *in vitro*. We have not performed assays of drug concentrations in serum or the brain of animals. This prevents the development of conclusions regarding the effects of these drug combinations during the course of more prolonged or more severe infections. The results observed *in vitro* and in animal models could however be seen as complementary. In our study, both approaches showed anti-trypanosomal activity of posaconazole.

Posaconazole has proved to be safe and efficacious as antifungal treatment, included in immunocompromised patients [68] and when used at high dosage [69]. Posaconazole is an inhibitor of Cyp 3A4 enzyme but does not serve as a substrate, and thus has a moderate propensity for drug-drug interactions [70]. It was initially developed as an oral suspension that has shown limitations with respect to fasting state absorption [71,72]. A more recently approved delayed-release oral tablet could reduce inter-patient variability and can be taken once daily without the need for food intake to support adequate absorption [73,74]. Data concerning posaconazole diffusion into the brain are limited [75,76], although efficacy when treating invasive cerebral fungal infections have been reported in murine models [77] and clinical cases reports, suggesting that it crosses the blood brain barrier in sufficient quantities to treat cerebral fungal infection and thus possibly treat late stage sleeping sickness [78–80]. The recent results in a phase II clinical trial with posaconazole for Chagas disease have been disappointing [81]. In that trial, posaconazole was proposed as a monotherapy. While the parasitemia dropped below detection limit after treatment, it was observed that 10 months later more patients in the posaconazole groups had treatment failure during follow-up than in the comparative benznidazole group. Thereafter, some authors have proposed not to abandon the triazoles during Chagas disease but to find a suitable partner for combination therapy [82]. Our results suggest that this approach could be considered for HAT with an eflornithine-based combination. Indeed, posaconazole is considered as well tolerated unlike nifurtimox, which has a significant toxicity, particularly when used alone in high doses [83]. Thus, posaconazole-eflornithine combination (PECT) could be considered as a potential alternative to arsenical treatment for trypanosomiasis with, compared to NECT, expected improved tolerance. Additional research should be performed to determine potential combinations with other drugs currently under investigation in HAT such as fexinidazole [84,85] or oxaboroles [86]. Further research should also be conducted to identify new molecules that target more specifically trypanosome CYP51 with enhanced affinity to the enzyme in order to improve the efficiency in combination [87].

## Supporting Information

S1 Table Genotype characterization of the *T*. *b*. *gambiense Feo* strain. (DOCX)

S1 References Supporting Information References. (DOCX)

S1 Text Genotype characterization of *T*.*b*. *gambiense* Feo strain. (DOCX)
