# Correction: *Trypanosoma brucei* CYP51: Essentiality and Targeting Therapy in an Experimental Model

**DOI:** 10.1371/journal.pntd.0005328

**Published:** 2017-01-31

**Authors:** The PLOS Neglected Tropical Diseases Staff

There are errors in the affiliations. The complete, correct affiliations are as follows:

Frédéric-Antoine Dauchy^1,2,3^, Mélanie Bonhivers^4,5^, Nicolas Landrein^4,5^, Denis Dacheux^4,5,6^, Pierrette Courtois^1,2^, Florian Lauruol^1,2^, Sylvie Daulouède^1,2^, Philippe Vincendeau^1,2,7^, Derrick R. Robinson^4,5^

1 University of Bordeaux, UMR IRD CIRAD INTERTRYP 177, laboratoire de parasitologie, F-33000 Bordeaux, France.

2 IRD-CIRAD-University of Bordeaux, UMR INTERTRYP 177, F-34394 Montpellier, France.

3 University Hospital of Bordeaux, Department of infectious and tropical diseases, Hôpital Pellegrin, F-33076 Bordeaux, France.

4 University of Bordeaux, Microbiologie Fondamentale et Pathogénicité, UMR 5234, F-33000 Bordeaux, France.

5 CNRS, Microbiologie Fondamentale et Pathogénicité, UMR 5234, F-33000 Bordeaux, France.

6 Bordeaux INP, ENSTBB, Microbiologie Fondamentale et Pathogénicité, UMR 5234, F-33000 Bordeaux, France.

7 University Hospital of Bordeaux, laboratoire de parasitologie, Hôpital Pellegrin, F-33076 Bordeaux, France.
