# Supplementary material for: Trypanosoma brucei CYP51: Essentiality and Targeting Therapy in an Experimental Model
Source: PLoS Negl Trop Dis. 2016 Nov 17;10(11):e0005125. doi: 10.1371/journal.pntd.0005125 (PMC5113867; doi:10.1371/journal.pntd.0005125)
Supplement: S1 Table — (DOCX) [file pntd.0005125.s001.docx]

**Table S1: Genotype characterization of the *T. b. gambiense Feo* strain.**

| Identification | Focus | Year | Host | Isolate Ref. | Genotyping Ref. | Microsatellites (Repetition number) | | | | |
| --- | --- | --- | --- | --- | --- | --- | --- | --- | --- | --- |
|  |  |  |  |  |  | **MORF2-CA** | **M6C8-CA** | **MT3033-AC/TC** | **MST19-TA/CA** | **PE procyclin** |
|  |  |  |  |  |  | **Allele 1-2** | **Allele 1-2** | **Allele 1-2** | **Allele 1-2** | **Allele 1-2-3-4-5** |
| *Trypanosoma bruceigambiense* Type I | | | | | | | | | | |
| 1972 | Ivory Coast | 1997 | Human | [2] | [2], This study | 16 - 16 | 13 - 53 | 30 - 48 | 22 - 22 | 15 - 20 - 21 - 24 |
| 1135 | Ivory Coast | 1991 | Human | [3] | [3] | 16 - 16 | 13 - 44 | 30 - 44 | 22 - 22 | 15 - 20 - 21 |
| LiTat-1/1 | Ivory Coast | 1952 | Human | [4] | [2] | 16 - 16 | 13 - 44 | 30 - 43 | 22 - 22 | 15 - 19 - 20 - 21 |
| 1122 | Ivory Coast | 1991 | Human | [3] | [3] | 16 - 16 | 13 - 44 | 30 - 44 | 22 - 22 | 15 - 20 - 21 |
| TB26 | Congo | 1983 | Pig | [5-7] | [2] | 16 - 16 | 13 - 37 | 30 - 30 | 22 -22 | N/D |
| *Trypanosomabruceigambiense* Type II | | | | | | | | | | |
| Feo | Togo | 1961 | Human | [5,8] | This study | 57 - 57 | 69 - 70 | 17 - 17 | 21 - 34 | 20 - 21 - 25 |
| HTAG/107-1 | Ivory Coast | 1986 | Human | [9-11] | [2] | 13 - 48 | 23 - 37 | 14 - 14 | 24 - 25 | 21 - 22 - 23 – 26 - 28 |
| TH2 (78E) | Ivory Coast | 1978 | Human | [10,12] | [2] | 13 - 49 | 38 - 48 | 19 - 22 | 31 - 40 | 22 - 23 – 26 - 28 |
| 2178 | Ivory Coast | 1997 | Pig | [2] | [2], This study | 12 - 43 | 31 - 35 | 14 - 28 | 24 - 31 | 20 - 21 - 22 - 29 - 30 |
| 2171 | Ivory Coast | 1997 | Pig | [2] | [2] | 48 - 49 | 29 - 46 | 15 - 15 | 25 - 25 | N/D |

The genotype of Feo strain was analyzed as described (2) by determining the number of repeats per allele and compared to group 1 and group 2 genotypes. Isolate 1972 and 2178 have been used as controls for group 1 and 2 respectively. N/D: not determined.
