# Supplementary material for: Trypanosoma brucei CYP51: Essentiality and Targeting Therapy in an Experimental Model
Source: PLoS Negl Trop Dis. 2016 Nov 17;10(11):e0005125. doi: 10.1371/journal.pntd.0005125 (PMC5113867; doi:10.1371/journal.pntd.0005125)
Supplement: S1 References — (DOCX) [file pntd.0005125.s002.docx]

**Supplemental References**

1. Clayton CE, Fueri JP, Itzhaki JE, Bellofatto V, Sherman DR, et al. (1990) Transcription of the procyclic acidic repetitive protein genes of Trypanosoma brucei. Mol Cell Biol 10: 3036–3047.
2. Biteau N, Bringaud F, Gibson W, Truc P, Baltz T (2000) Characterization of Trypanozoon isolates using a repeated coding sequence and microsatellite markers. Mol Biochem Parasitol 105: 187–202.
3. Giroud, C., Ottones, F., Coustou, V., Dacheux, D., Biteau, N., Miezan, B., Van Reet, N., Carrington, M., Doua, F., and Baltz, T. (2009). Murine models for Trypanosoma brucei gambiense disease progression from silent to chronic infections and early brain tropism. PLoSNegl. Trop. Dis. 3, e509.
4. Baltz T, Baltz D, Giroud C, Crockett J. Cultivation in a semi-defined medium of animal infective forms of Trypanosoma brucei, T. equiperdum, T. eTansi, T. rhodesiense and T. gambiense. Embo J 1985;4:1273 – 7.
5. Tait A, Babiker EA, Le Ray D. Enzyme variation in Trypanosoma brucei spp. I. Evidence for the subspeciation of Trypanosoma bruceigambiense. Parasitology 1984;89:311 – 26.
6. Kanmogne GD, Stevens JR, Asonganyi T, Gibson WC. Genetic heterogeneity in the Trypanosoma brucei gambiense genome analysed by random amplification of polymorphic DNA. Parasitol Res 1996;82:535 – 41.
7. Truc P, Tibayrenc M. Population genetics of Trypanosoma brucei in central Africa: taxonomic and epidemiological significance. Parasitology 1993;106:137–49.
8. Paindavoine P, Pays E, Laurent M, Geltmeyer Y, Le Ray D, Mehlitz D, Steinert M: The use of DNA hybridization and numerical taxonomy in determining relationships between Trypanosoma brucei stocks and subspecies. Parasitology. 1986, 92: 31-50.
9. Stevens JR, Lanham SM, Allingham R, Gashumba JK. A simplified method for identifying subspecies and strain groups in Trypanozoon by isoenzymes. Ann Trop Med Parasitol 1992;86:9–28.
10. Bromidge T, Gibson W, Hudson K, Dukes P. Identification of Trypanosoma brucei gambiense by PCR amplification of variant surface glycoprotein genes. Acta Trop (Basel) 1993;53:107–19.
11. Penchenier L, Mathleu-Daude F, Brengues C, Banuls AL, Tibayrenc M. Population structure of Trypanosoma brucei S. L. in Cote d’Ivoire assayed by multilocus enzyme electrophoresis: epidemiological and taxonomical considerations. J Parasitol 1997;83:19–22.
12. Mehlitz D, Zillmann U, Scott CM, Godfrey DG. Epidemiological studies on the animal reservoir of Gambiense sleeping sickness. Part III. Characterization of Trypanozoon stocks by isoenzymes and sensitivity to human serum. Tropenmed Parasitol 1982;33:113-8.
