# Supplementary material for: Trypanosoma brucei CYP51: Essentiality and Targeting Therapy in an Experimental Model
Source: PLoS Negl Trop Dis. 2016 Nov 17;10(11):e0005125. doi: 10.1371/journal.pntd.0005125 (PMC5113867; doi:10.1371/journal.pntd.0005125)
Supplement: S1 Text — (DOCX) [file pntd.0005125.s003.docx]

**Supplemental Information**

**Genotype characterization of *T.b. gambiense* Feo strain**

Microsatellite analysis of the MORF2-CA, M6C8-CA, MT3033-AC/TC and MEST19-AT/GT loci and minisatellite analysis of the PARP locus (PE procyclin repetition) (1) were performed as previously described (2, 3). Briefly, after PCR, the amplified products were first analyzed on a 1.5 agarose gel and were shipped to Eurofins Genomics (Ebersberg, Germany) for fragment analysis using standard procedures of capillary electrophoresis. The number of repeats at each locus was calculated based on the size of the PCR product (2). We could identify with markers genotype: MEST19, 21/34; PE procyclin A, 20, 21, 22, 29, 30; MT3033,17/17; MORF2, 57/ 57; M6C8, 69/70 that the *T. b. gambiense* Feo strain belongs to group 2 *gambiense* as reported in the table S1.
